# A Hetero-Photoautotrophic Two-Stage Cultivation Process for Production of Fucoxanthin by the Marine Diatom *Nitzschia laevis*

**DOI:** 10.3390/md16070219

**Published:** 2018-06-25

**Authors:** Xue Lu, Han Sun, Weiyang Zhao, Ka-Wing Cheng, Feng Chen, Bin Liu

**Affiliations:** 1 Institute for Food & Bioresource Engineering, College of Engineering, Peking University, Beijing 100871, China; gracexuelu@pku.edu.cn (X.L.); shlyg2242@163.com (H.S.); 1501214672@pku.edu.cn (W.Z.); 2 BIC-ESAT, College of Engineering, Peking University, Beijing 100871, China; 3 Institute for Advanced Study, Shenzhen University, Shenzhen 518060, China; albertkw.cheng@gmail.com

**Keywords:** microalgae, diatom, *Nitzschia laevis*, fucoxanthin, fed-batch, mixed lights

## Abstract

There is currently much interest in fucoxanthin due to its broad beneficial health effects. The major commercial source of fucoxanthin is marine seaweed, which has many shortcomings, and has thus restricted its large-scale production and more diversified applications. In this study, growth characteristics and fucoxanthin accumulation were evaluated to explore the potential of the marine diatom *Nitzschia laevis* in fucoxanthin production. The results suggested that heterotrophic culture was more effective for cell growth, while the mixotrophic culture was favorable for fucoxanthin accumulation. A two-stage culture strategy was consequently established. A model of exponential fed-batch culture led to a biomass concentration of 17.25 g/L. A mix of white and blue light significantly increased fucoxanthin content. These outcomes were translated into a superior fucoxanthin productivity of 16.5 mg/(L·d), which was more than 2-fold of the best value reported thus far. The culture method established herein therefore represents a promising strategy to boost fucoxanthin production in *N. laevis*, which might prove to be a valuable natural source of commercial fucoxanthin.

## 1. Introduction

Fucoxanthin is one of the primary carotenoids in marine brown seaweeds, diatoms and golden algae, and it plays a major role in the light-harvesting complex of photosystems by forming a complex with the chlorophyll a/c binding proteins [1,2]. It has been reported to exhibit an array of beneficial biological activities including anti-obesity, anti-diabetes, anti-cancer, anti-allergy, anti-inflammation, anti-oxidation, and anti-osteoporosis [3,4]. Hence, fucoxanthin has great potential for application in pharmaceutical, cosmetic, food and aquaculture industries [5,6].

Currently, commercially available fucoxanthin is mainly extracted from marine seaweeds [7,8]. However, the use of marine seaweeds as a natural source of fucoxanthin has important shortcomings, including low growth rate, low fucoxanthin content, poor product quality and potential negative impact on the environment [9,10]. These shortcomings have compromised the development of cost-effective production methods. In contrast, many microalgae, especially diatoms, produce fucoxanthin as one of their main cellular pigments with contents ranging from 0.22% to 2.66% of dry cell weight, which could be up to 100 times higher than that in brown seaweeds. In addition, they grow rapidly and perform robustly in bioreactors. Therefore, these microalgae might prove to be much better producers of fucoxanthin than brown seaweeds [7].

Although many microalgae are rich in fucoxanthin, their fucoxanthin productivity is often poor. Fucoxanthin-rich microalgae, including *Mallomonas* sp., *Isochrysis galbana*, *Phaeodactylum tricornutum* and *Odontella aurita*, have been reported to have high fucoxanthin content (1.65% to 2.66%), but the maximum fucoxanthin productivity only ranged from 1.75 to 7.96 mg/(L·d) [11,12,13,14]. The low productivity of fucoxanthin in these previous studies was likely due to inefficient utilization of organic carbon source(s) in the culture medium [15]. Hence, it would be of great importance to explore fucoxanthin-rich microalgae that can grow to a high cell density under heterotrophic conditions. Nevertheless, there has been very limited research on heterotrophic production of fucoxanthin by microalgae [7].

Although the marine diatom *Nitzschia laevis* can grow photoautotrophically, large-scale production of fucoxanthin will be hindered by an inability to obtain high cell densities in photobioreactors due to light limitation. However, heterotrophic culture eliminates the requirement for light and offers the potential of elevating biomass concentration and productivity alternative to photoautotrophic growth [15]. *N. laevis* was found to be capable of growing and producing fucoxanthin heterotrophically [16]. *N. laevis* therefore has the potential to be a candidate for industrial production of fucoxanthin, which has been seriously underexploited [17]. In the present study, effects of various light intensities on cell growth and fucoxanthin accumulation in *N. laevis* were evaluated. After identifying the optimal light condition, a two-stage fed-batch cultivation strategy utilizing fermentors and bubbling column bioreactors was examined aiming to optimize biomass accumulation and fucoxanthin productivity. To the best of our knowledge, this has been the first study on the use of a two-stage cultivation strategy for the production of fucoxanthin by *N. laevis*, which achieved superior productivity.

## 2. Results

### 2.1. Effects of Light on Cell Growth of N. laevis

**Figure 1:**
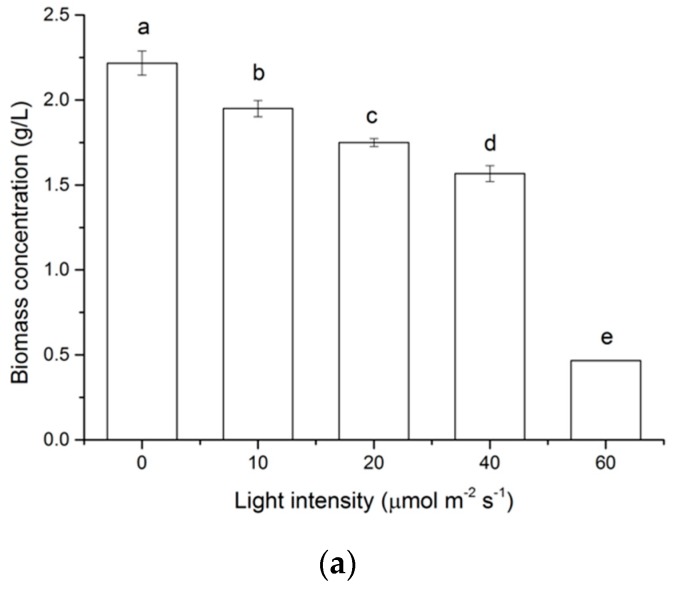

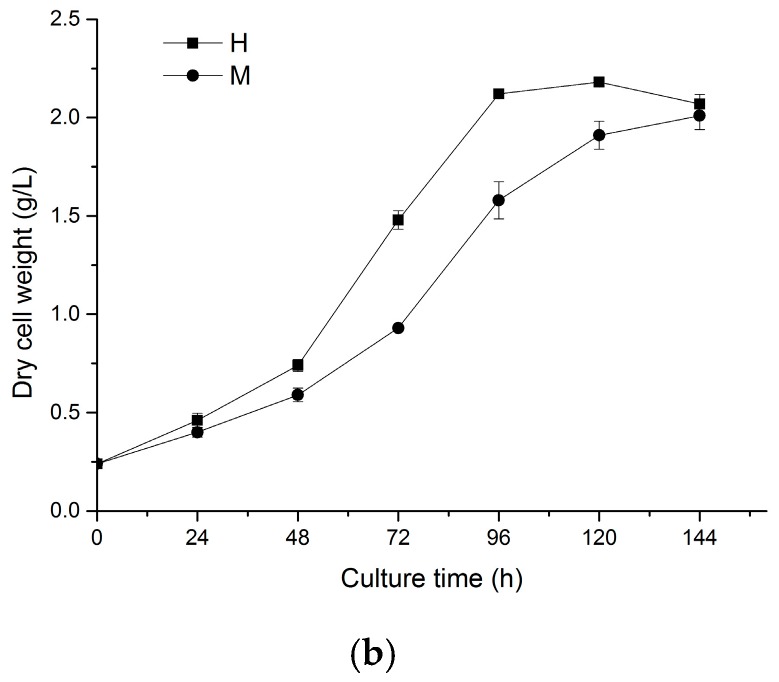
Effect of light on the growth of *N. laevis*. (**a**) biomass concentrations of *N. laevis* under different light intensities at 96 h; (**b**) growth curves of *N. laevis* in heterotrophic culture (H) and mixotrophic culture (M). Values are mean ± SD of at least three independent experiments. Bars not sharing a common letter (a, b, c, d, e) are significantly different (*p* < 0.05).

In the present study, various light intensities (0, 10, 20, 40, 60 μmol m^−2^ s^−1^) were supplied to examine the effect of light on the growth of *N. laevis*. As shown in Figure 1a, the highest biomass concentration of 2.22 g/L was obtained in darkness at 96 h. It gradually decreased as light intensity increased from 10 to 40 μmol m^−2^ s^−1^ and there was a more dramatic decrease when it changed from 40 to 60 μmol m^−2^ s^−1^. When the light intensity of the mixotrophic culture was set at 10 μmol m^−2^ s^−1^, the biomass concentration reached a maximum of 1.91 g/L at day 6 (Figure 1b). Although the growth pattern of the heterotrophic culture was similar to that of the mixotrophic culture, the specific growth rate in the dark of the former was much higher than that of the latter (0.024 ± 0.0021 h^−1^ vs. 0.0176 ± 0.0012 h^−1^).

### 2.2. Effects of Light on the Photosystem II of N. laevis

**Figure 2:**
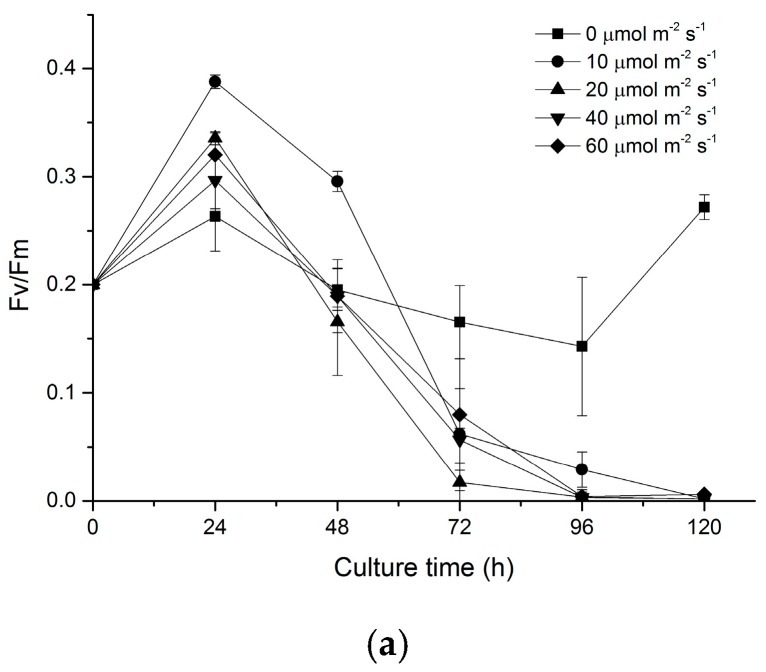

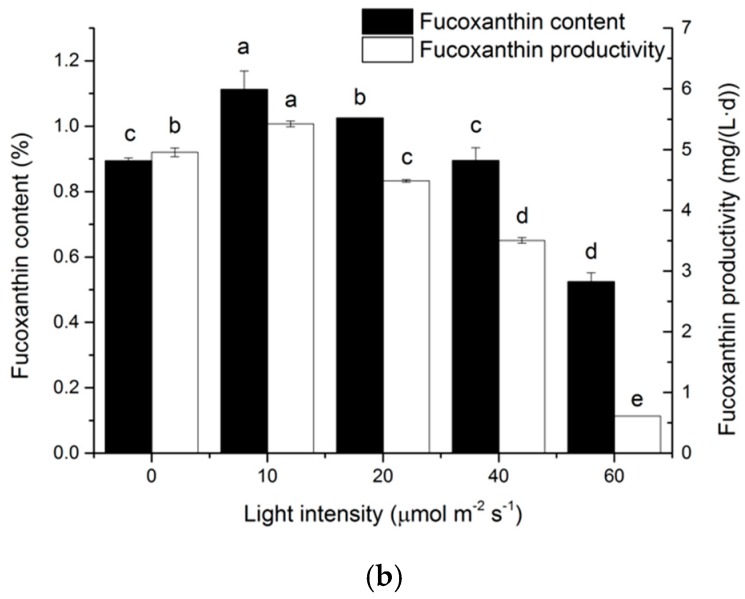
Effect of light on the *F*_v_/*F*_m_ and fucoxanthin accumulation of *N. laevis*. (**a**) changes of *F*_v_/*F*_m_ with culture time on different light intensities; (**b**) fucoxanthin contents and productivities of different light intensities at 96 h. Values are mean ± SD of at least three independent experiments.

Chlorophyll fluorescence parameter *F*_v_/*F*_m_ reflects the photosynthesis activity of photosystem II (PS II). In order to understand the pattern of biomass accumulation of *N. laevis*, *F*_v_/*F*_m_ was monitored using a Water-PAM (pulse-amplitude modulation) fluorometer. It was observed that *F*_v_/*F*_m_ values of mixotrophic and heterotrophic cells increased dramatically (at 24 h) when they were inoculated in fresh medium (Figure 2a). Mixotrophic cells showed the highest increase of *F*_v_/*F*_m_ at a light intensity of 10 μmol m^−2^ s^−1^, while heterotrophic cells (0 μmol m^−2^ s^−1^) showed the lowest increase. After 24 h, *F*_v_/*F*_m_ of the cells under mixotrophic conditions decreased significantly with time. However, there was no significant change from 24 to 96 h when the cells were cultured under heterotrophic conditions.

### 2.3. Effects of Light Intensity on Fucoxanthin Accumulation of N. laevis

Although cell growth of *N. laevis* with exogenous glucose was inhibited by light, fucoxanthin accumulation was elevated significantly. In heterotrophic cells, the fucoxanthin content was 0.89% (of cell dry weight). It is worth noting that the fucoxanthin contents of the cells cultured under light intensity of 10 μmol m^−2^ s^−1^ and 20 μmol m^−2^ s^−1^ reached 1.11% and 1.03%, which were 24.42% and 14.64% higher than those cultured in darkness, respectively (Figure 2b). However, at higher light intensity (60 μmol m^−2^ s^−1^), the fucoxanthin content decreased dramatically to only 0.52%. From the dry weight point of view, the presence of moderate light intensity improved the productivity of fucoxanthin. The fucoxanthin productivity of cells cultured for 96 h with 10 μmol m^−2^ s^−1^ light reached 5.42 mg/(L·d), which was 9.27% higher than that of cells cultured in darkness.

### 2.4. Effects of Light Composition on Fucoxanthin Accumulation in N. laevis

**Figure 3:**
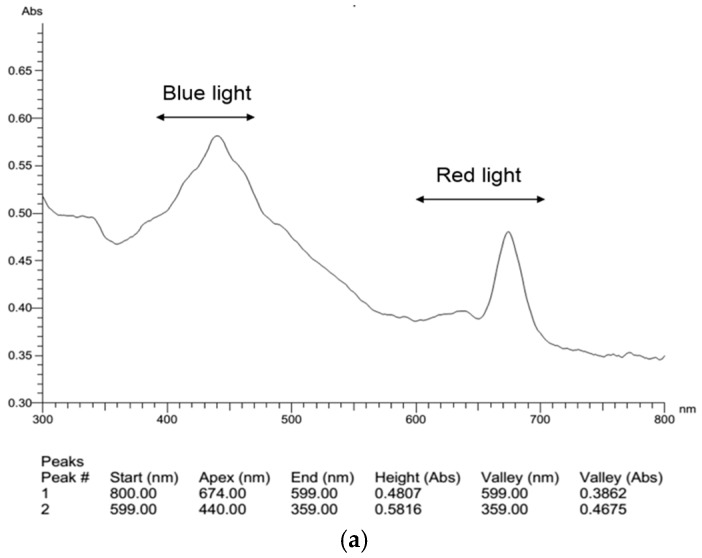

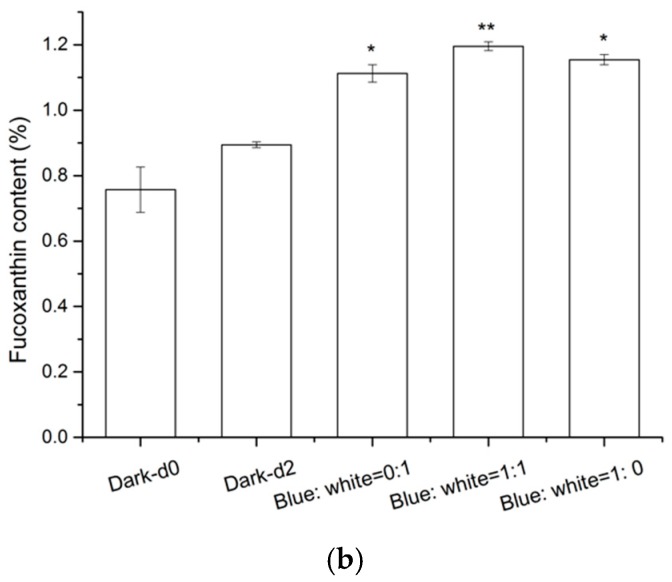
Effects of light quality on *N. laevis*. (**a**) full-wavelength scan of *N. laevis*; (**b**) fucoxanthin content under different lights. Values are mean ± SD of at least three independent experiments. * *p* < 0.05; ** *p* < 0.01.

As mentioned above, high-intensity light inhibited the growth of *N. laevis* but promoted fucoxanthin generation. To explore if fucoxanthin biosynthesis could be enhanced by manipulating the wavelength of light, a full-wavelength scan of this diatom was carried out. As shown in Figure 3a, there were two absorption peaks at 440 nm and 674 nm. The peak at 674 nm generally corresponds to absorption of red light, which was related to chlorophyll content. On the other hand, the peak at 440 nm generally corresponds to the absorption of blue light, which has been demonstrated to be involved in the synthesis of carotenoids, including fucoxanthin [18]. Hence, LED light of white, blue and a mixture of the two (1:1) were used following heterotrophic culture of *N. laevis* for two days.

After light treatment (10 μmol m^−2^ s^−1^) for two days, the carotenoid content of the mixotrophic culture was found to be higher than that of the heterotrophic culture, and the highest carotenoid content (8.34%) was obtained with blue light (Figure S2). However, the highest carotenoid productivity of 137 mg/(L·d) was achieved under the mixed light condition. Different from total carotenoids, the fucoxanthin content under mixed light was 1.20% of cell dry weight, which was significantly higher than that obtained under monochrome light (Figure 3b). In accordance with the trends shown in Figure 1a, the mixotrophic culture resulted in a 33.7% higher content of fucoxanthin than the heterotrophic culture, which indicates that light, especially a mix of blue and white (1:1), could promote the accumulation of fucoxanthin in *N. laevis*.

### 2.5. Two-Stage Cultivation Strategy for Fucoxanthin Production by N. laevis

As stated above, although both light intensity and composition exhibited significant effects on fucoxanthin biosynthesis in *N. laevis*, heterotrophic culture was more suitable for biomass accumulation. Therefore, a two-stage cultivation strategy, including high cell density fermentation in darkness and stimulation of fucoxanthin synthesis with moderate light, was established.

**Figure 4:**
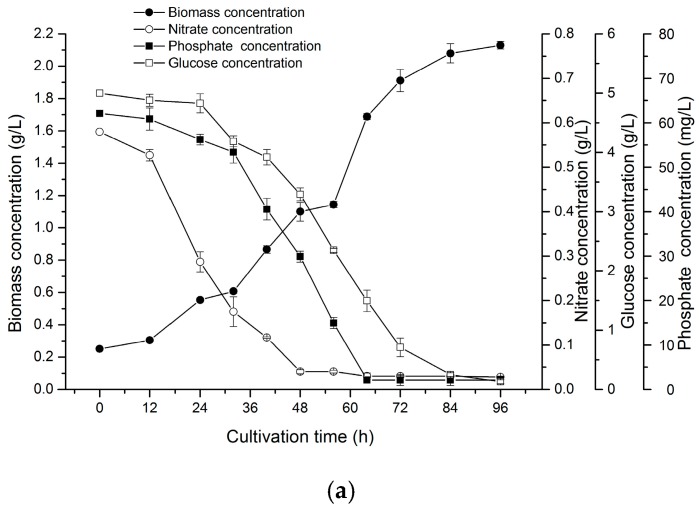

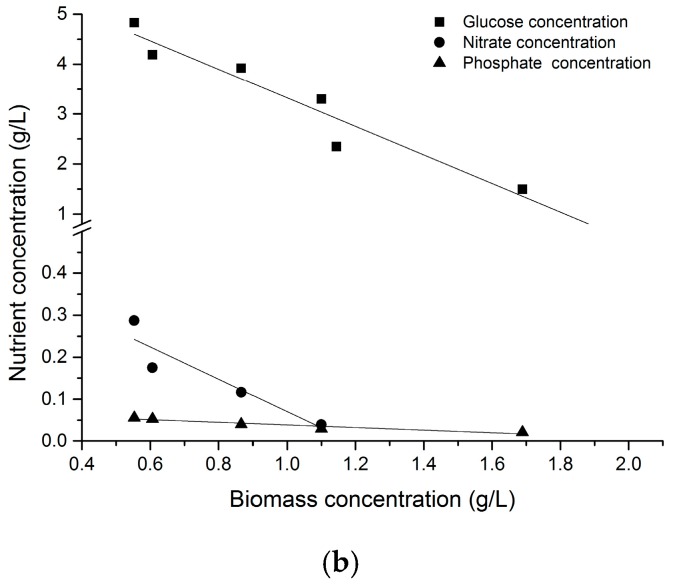
Relationship between biomass concentrations and nutrient concentrations. (**a**) relationships among biomass concentration, glucose concentration, nitrate concentration and phosphate concentration in the culture period; (**b**) biomass of *N. laevis* on glucose, nitrate and phosphate in batch culture. Values are mean ± SD of at least three independent experiments.

Up to now, the effects of various nutrients on the growth of *N. laevis* remain poorly understood. The data (Figure 4a) from our evaluation of the relationship between biomass accumulation and main nutrient consumptions revealed that after a short lag phase, *N. laevis* grew rapidly from 24 h to 72 h, and then its growth rate gradually decreased and reached a maximum biomass of 2.13 g/L at 96 h. Biomass accumulation exhibited respectively a linear correlation with the consumption of glucose, nitrate and phosphate in the exponential phase. The biomass yields on glucose, nitrate and phosphate were 0.35 g/g, 2.60 g/g and 32.21 g/g, respectively. Subsequently, a fed-batch model was established to feed the culture during the exponential phase aiming to optimize biomass accumulation.

Based on the biomass yields on the nutrients in the batch cultivation of *N. laevis*, the mass ratio of glucose:nitrate:phosphate in the feeding medium was determined to be 91.55:12.37:1.00. Equation (6) was applied to calculate the feeding volume. The specific growth rate in the batch culture was determined to be *μ* = 0.0276 h^−1^, which was used as the basis for determination of the coefficients of the feeding equations. For flask culture, the initial conditions were set as follows: *C*_0_ = 0.25 g/L, *V*_0_ = 100 mL, *F*_G_ = 500 g/L. Thus, Equation (6) was simplified to

(1) $$V_{F\cdot s}=0.004\exp^{\left(0.0276t\right)}.$$

For fermentor culture, the initial conditions were set as follows: *C*_0_ = 0.32 g/L, *V*_0_ = 1200 mL, *F*_G_ = 500 g/L. Thus, Equation (6) was simplified to

(2) $$V_{F\cdot s}=0.060\exp^{\left(0.0276t\right)}.$$

**Figure 5:**
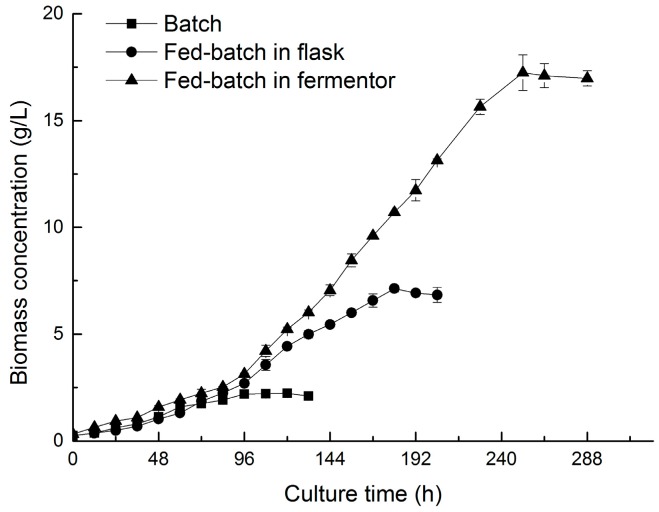
Effect of fed-batch on the growth of *N. laevis* according to the feeding model. Values are mean ± SD of at least three independent experiments.

As shown in Figure 5, the biomass of *N. laevis* in flasks by the fed-batch method entered a nearly steady state after 180 h, which might be due to a lack of dissolved oxygen caused by the high biomass concentration (7.13 g/L). In the 3.0-L fermentor, a peak biomass concentration of 17.25 g/L was achieved at 252 h, which was 7.7-fold of that achieved with the batch culture (2.22 g/L). These data suggest that fed-batch culture with the above feeding model might be a promising strategy to enhance biomass accumulation in heterotrophic fermentation of *N. laevis*. Subsequently, blue-white (1:1) mixed light was applied to the two-stage culture to induct fucoxanthin accumulation. The final fucoxanthin productivity reached 16.5 mg/(L·d), which has been the highest reported thus far (Table 1). The two-stage cultivation strategy established herein therefore provides an effective means to enhance fucoxanthin production by *N. laevis*.

## 3. Discussion

Many studies have demonstrated that heterotrophic culture of certain microalgae could lead to higher biomass concentrations than mixotrophic and photoautotrophic culture. In agreement with previous reports, our results also indicate that light has a negative impact on biomass accumulation and cell growth as reflected by a lower final cell density and lower specific growth rate in mixotrophic culture compared with heterotrophic culture. Heterotrophic culture obtained the highest biomass concentration of *Spirulina* sp. as compared to mixotrophic and photoautotrophic growth modes [24]. Heredia-Arroyo et al. also demonstrated that heterotrophic culture of *Chlorella protothecoides* grew better than mixotrophic culture [25]. This phenomenon was probably due to the availability of organic carbon source(s), which provided sufficient energy and intermediates to support cell metabolism, thus promoting cell enlargement and division. In the present investigation, heterotrophic cultivation of *N. laevis* resulted in the highest biomass concentration, and application of light attenuated the promoting effect on biomass concentration. The energy and substrate sources from photosynthesis and glucose absorption were suggested as the competitive pathways for subsequent metabolism, and glucose absorption was the better source for its significantly stimulating effect on cell growth. These results might be attributed to cell damage induced by light under the glucose-repletion condition.

*F*_v_/*F*_m_ represents the maximum quantum yield of primary PSII and is generally accepted as a measure for the PSII status. Our result was in line with a previous study which found that *F*_v_/*F*_m_ of a mixotrophically cultured diatom decreased from 0.6 to 0.1 when the cells were exposed to low or high intensity of light [26]. The observed decrease in *F*_v_/*F*_m_ provided indirect evidence for degradation of the photosynthetic apparatus, which indicated that mixotrophic culture likely damaged the photosystem of *N. laevis* [26]. A similar trend was also observed for the ROS levels in the cells (Figure S1). The elevated ROS levels induced by light may damage the *N. laevis* cells and affect their growth. We hypothesized that the photosynthetic activity of PS II might correspond to cell growth and final biomass concentration of the diatom. Consistent with our hypothesis, heterotrophic cells maintained the highest photosynthetic activity of PS II even after 72 h, and the highest biomass concentration was obtained under heterotrophic conditions. Of note, under mixotrophic conditions, light intensity as low as 10 μmol m^−2^ s^−1^ was already able to support a high *F*_v_/*F*_m_ and resulted in a high cell density. Feng et al. also found that the *F*_v_/*F*_m_ of *Isochrysis* cells of exhibited a drastic decline with a decrease of biomass concentration [20].

Fucoxanthin, a xanthophyll, is an accessory pigment in the chloroplasts of diatoms. It absorbs light primarily in the blue-green to yellow-green region of the visible spectrum. Our results here are in agreement with previous studies which also showed that moderate light intensity favors the synthesis of fucoxanthin [27]. Our study has demonstrated that light had dual effects on fucoxanthin biosynthesis in *N. laevis.* At low light intensity, fucoxanthin accumulation was enhanced. When light intensity was increased beyond a certain level, fucoxanthin synthesis was impaired. It was suggested that the variation in fucoxanthin content caused by light might be related to modulation of the Diadinoxanthin Cycle [14], whereby, diatom cells tend to convert the precursors to diatoxanthin (instead of fucoxanthin) in order to adapt to the abiotic stress under high intensity light. The beneficial effect of low light intensity on fucoxanthin production and accumulation was also observed in the diatom *Cyclotella cryptica* in our previous work [7].

It has been reported that monochromatic light (especially blue light) had positive effects on microalgal cell growth and carotenoid accumulation [28]. Sun et al. also showed enhanced accumulation of astaxanthin with extra blue light in the green microalga *Haematococcus pluvialis* [29]. In addition, it has been found that blue light induced carotenoid synthesis in the bacteria *Myxocococcus xanthus* [30].

Development of a suitable feeding strategy is of critical importance to avoid potential detrimental effect on cell growth and product synthesis caused by overfeeding or underfeeding of nutrients in fed batch culture. To this end, a simple yet accurate model capable of predicting cell growth and nutrient utilization during fed-batch fermentation was adopted to design an exponential feeding strategy [31]. According to the relationship between nutrient consumption and biomass accumulation, the final feeding model for *N. laevis* was determined to be: $V_{F\cdot S}=0.004\exp^{0.0276t}$ and $V_{F\cdot S}=0.060\exp^{0.0276t}$ for flask and fermentor culture, respectively. The biomass (17.25 g/L) and specific growth rate (0.0276 h^−1^) achieved represent significant improvement over those reported in the literature thus far. Furthermore, the fed-batch exponential feeding model established in this study is easy to operate and has low requirements for equipment and space. Furthermore, these advantages may help overcome some of the major problems associated with traditional fed-batch culture, namely feeding time and amounts of feeding nutrients. The feeding model might also be a useful tool in process optimization and control.

Biomass concentration and fucoxanthin content are key factors for fucoxanthin production by microalgae. Large-scale production of fermentation products is often limited by suboptimal biomass accumulation and productivity in conventional photoautotrophic systems [32,33]. Many studies have demonstrated that heterotrophic and mixotrophic culture of certain microalgae could lead to higher biomass concentrations than photoautotrophic culture [33]. Light illumination is an effective strategy of inducing microalgal cells to accumulate pigments such as astaxanthin and fucoxanthin. However, after a long-term cultivation in the darkness, the microalgal cellular photosynthesis systems of cells, especially cells at stationary phase, were not ready for light irradiation cells due to their low chlorophyll contents [34]. Thus, *N. laevis* cells at the end of the exponential phase were harvested for light induction in the present study. The observation led us to explore the feasibility to increase the fucoxanthin productivity with a two-stage cultivation model. The results supported our hypothesis that heterotrophic culture of *N. laevis* produced a high biomass with high fucoxanthin productivity. Although some microalgae were reported to have higher fucoxanthin contents than *N. laevis*, their low biomass concentrations significantly compromised productivity of fucoxanthin.

## 4. Materials and Methods

### 4.1. Algal Strain and Culture Conditions

*Nitzschia laevis* UTEX 2047 was purchased from the Culture Collection of Algae at The University of Texas at Austin, TX, USA. *N. laevis* cells were maintained in liquid LDM medium and subcultured in the dark.

The LDM medium consisted of 1 g tryptone, 892 mL artificial seawater, 100 mL Bristol solution, 6 mL PIV metal solution, 1 mL stock solution of biotin (25.0 × 10^−5^ g /L) and 1 mL vitamin B_12_ (15.0 × 10^−5^ g/L). All the fermentation media were supplemented with 120 mg/L Na_2_SiO_3_·9H_2_O, 0.68 g/L NaNO_3_ and 5 g/L glucose. The media were adjusted to pH 8.5 and autoclaved at 121 °C for 20 min [19].

The cell suspensions were centrifuged at 3000 *g* for 5 min, and the cell pellets were washed twice with distilled water before transferring into 250-mL Erlenmeyer flasks each containing 100 mL of fresh LDM medium. Pre-sterilized glucose stock solution was added to the medium at a final concentration of 5 g/L. The cell cultures were incubated at 23 °C with orbital shaking at 150 rpm. The mixotrophic cultures were continuously illuminated with light emitting diode (LED) lights, whereas the heterotrophic culture was kept in darkness.

The effect of light intensity on cell growth and fucoxanthin content was evaluated in flasks with initial light intensity set at 0, 10, 20, 40 and 60 μmol m^−2^ s^−1^, respectively. Then, white, blue and a mix of the two (1:1 ratio) were used to evaluate their respective effects on cell growth and pigments biosynthesis. Heterotrophic cells from 3-L fermentors (BioFlo 115, New Brunswick, NJ, USA) were stimulated with the mixed light in 200-mL glass bubble column (3-cm diameter) photobioreactors for 24 h.

### 4.2. Feeding Model of Fed-Batch Culture

The feeding model was based on the substrate consumption model, which aimed at balancing the concentration of nutrients. The equation could be expressed as:

(3) $$\frac{dC_{s}}{dt}=C_{F\cdot s}-\frac{1}{Y_{xs}}\frac{dC_{x}}{dt},$$

where *C_s_* and *C_x_* are the concentration of substrate and cells (g/L), respectively; *C_F·s_* is the feeding concentration of substrate (g/mL/h); and *Y_xs_* is the yield of biomass on a nutrient (g/g).

Since $dC_{s}/dt=0$ when at equilibrium, Equation (3) could be simplified as

(4) $$C_{F\cdot s}=\frac{1}{Y_{xs}}\mu C_{x}$$

and

(5) $$C_{F\cdot s}=\frac{1}{Y_{xs}}\mu C_{0}\exp^{\left(\mu t\right)},$$

where *μ* is the specific growth rate (h^−1^); and *C*_0_ is the initial biomass (g/L). To facilitate the operation of the fed-batch culture, the volume was used as the feeding index. Thus, Equation (5) becomes

(6) $$V_{F\cdot s}=\frac{\frac{1}{Y_{xs}}\mu V_{0}C_{0}\exp^{\left(\mu t\right)}}{F_{s}},$$

where *V*_0_ is the initial culture volume (mL); *F_s_* is the concentration of substrate in fed-batch culture (g/L); and *V_F·s_* is the feeding rate (mL/h). Fed-batch culture of *N. laevis* was carried out in both Erlenmeyer flasks and fermenters by feeding nutrients at regular time intervals and was compared with a batch culture without addition of nutrients. The fed-batch stock medium consisted of glucose 500.0, NaNO_3_ 113.4, and KH_2_PO_4_ 16.6 (g/L). Cells in 3.0 L fermentation broth were transferred to 200 mL bubble column photobioreactors and treated with a mixture of white and blue (1:1) LED light for 2 days. Light intensity was set at 10 μmol m^−2^ s^−1^.

### 4.3. Determination of Biomass Concentration

Five milliliters of cell suspension was centrifuged at 5000 *g* for 3 min. Then, the cell pellet was washed twice with distilled water and filtered through a pre-weighed filter paper (Whatman GF/C). The cells on the filter paper were dried to a constant weight at 80 °C in a vacuum oven.

The specific growth rate of *N. laevis* was calculated using Equation (7):

(7) $$\mu =\frac{\left(\ln N_{t}-\ln N_{0}\right)}{t},$$

where $N_{t}$ is the biomass concentration of the culture after *t* days (g/L); and $N_{0}$ is the initial biomass concentration (g/L).

### 4.4. Determination of Glucose, Nitrate and Phosphate Concentration

Glucose concentration of the supernatant was determined by DNS method [35]. Nitrate concentration of the supernatant was determined by measuring its optical density at 220 nm with a U-3900H spectrophotometer (Hitachi, Tokyo, Japan) [36]. Phosphate concentration of the supernatant was determined by measuring its optical density at 660 nm on a SpectraMax i3x Multi-Mode microplate reader (Molecular Devices, Sunnyvale, CA, USA).

### 4.5. Measurement of Quantum Yield of Photosystem II (PSII)

The maximum quantum yield (*F*_v_/*F*_m_) of photosystem II was determined by pulse-amplitude-modulated (PAM) fluorometry (Walz, Effeltrich, Germany). Three milliliters of cell suspension was incubated in the dark for 20 min. The dark-adapted minimum level of fluorescence (*F*_0_), the maximum level of fluorescence, and other chlorophyll fluorescence parameters were measured after a short pulse of high intensity light (*F*_m_) [37].

### 4.6. Measurement of Reactive Oxygen Species (ROS)

The intracellular ROS level was determined using an ROS assay kit (Beyotime Institute of Biotechnology, Shanghai, China) by measuring the oxidative conversion of 2′,7′-dichlorofluorescin diacetate (DCFH-DA) to a fluorescent compound dichlorofluorescin (DCF) according to the manufacturer’s instructions [35]. One milliliter of cell suspension was centrifuged at 13,500 *g* for 3 min and the cell pellet was resuspended in 500 μL of 10 μM DCFH-DA. After incubation for 20 min, the cells were washed two times with distilled water. The cell pellet was suspended in 1 mL of distilled water and fluorescence was measured on a microplate reader (Molecular Devices, Sunnyvale, CA, USA) with excitation wavelength at 488 nm and emission wavelength at 525 nm.

### 4.7. Carotenoid Analysis

For the analysis of pigment content, 1 mL of each cell suspension was centrifuged at 13,500 *g* for 3 min, and the cell pellets were suspended in 2 mL of methanol respectively. After incubation in the dark at 37 °C for 24 h, the extracts were centrifuged at 13,500 *g* for 3 min. Absorbance of the supernatants was measured at 480 nm. The pigment contents were calculated using the following equation [38]:

(8) $$\mathrm{Carotenoids}\;\left(\frac{\mu g}{\mathrm{mL}}\right)=4\times A_{480}.$$

The calculated pigment contents were converted to a dry weight basis and presented in mg/g.

### 4.8. Fucoxanthin Analysis

The lyophilized cell samples were ground and subsequently extracted with pure ethanol until the pellet was almost colorless. The ethanol layer was collected by centrifugation at 5000 *g* for 5 min and dried under a stream of nitrogen gas. Then, the dried extracts were re-dissolved in 1 mL of ethanol and filtered through a 0.22-μm Millipore membrane before subjecting to high performance liquid chromatography (HPLC) analysis [7].

### 4.9. Statistical Analysis

The data of all the measurements were obtained from three repetitions and analyzed using one-way analysis of variance (ANOVA) with subsequent post hoc multiple-comparison LSD tests, which were calculated using SPSS Statistics 18.0 software (IBM Corporation, Armonk, NY, USA).

## 5. Conclusions

In conclusion, the present investigation used high-density fermentation to produce fucoxanthin with broad beneficial health effects. By means of the two-stage cultivation strategy established in this study, heterotrophic culture together with mixed low light stimulation resulted in a superior fucoxanthin productivity, which has been the highest reported to date. Considering the encouraging findings, the diatom of *N. laevis* possesses good potential for industrial applications to produce fucoxanthin.

## Figures and Tables

**Table 1:** Biomass concentration, fucoxanthin content and fucoxanthin productivity of various microalgae.

Species		Biomass Concentration, g/L	Fucoxanthin Content, %	Fucoxanthin Productivity, mg/(L·d)	References
*Mallomonas* sp.		3.75	2.66	7.13	[19]
*Cyclotella cryptica*		1.72	1.29	3.38	[9]
*Chaetoceros calcitrans*			0.53		[20]
*Chaetoceros gracilis*				4.63	[21]
*Chaetoceros gracilis*			0.22		[10]
*Phaeodactylum tricornutum*		2.4	1.02	1.75	[19]
*Phaeodactylum tricornutum*		0.41	0.45	0.18	[22]
*Phaeodactylum tricornutum*		4.1	0.69	4.73	[23]
*Isochrysis aff. galbana*			1.82		[20]
*Odontella aurita*		6.36	2.17	7.96	[11]
*Nitzschia* sp.			0.49		[11]
*Nitzschi laevis *		0.2	0.5	0.17	[11]
*Nitzschi laevis *(batch in flask)		2.22	1.20	4.44	**This study**
*Nitzschi laevis *(fed-batch in flask)		7.13	1.20	9.01	**This study**
*Nitzschi laevis *(fed-batch in fermentor)		17.25	1.20	16.5	**This study**

## Supplementary Materials

The following are available online at http://www.mdpi.com/1660-3397/16/7/219/s1, Figure S1: Effect of light intensity on ROS level of *N. laevis*; Figure S2: Effect of light quality on carotenoids content of *N. laevis*.
