# Supplementary material for: A Hetero-Photoautotrophic Two-Stage Cultivation Process for Production of Fucoxanthin by the Marine Diatom Nitzschia laevis
Source: Mar Drugs. 2018 Jun 25;16(7):219. doi: 10.3390/md16070219 (PMC6070929; doi:10.3390/md16070219)
Supplement: Supplementary file 1 [file marinedrugs-16-00219-s001.pdf]

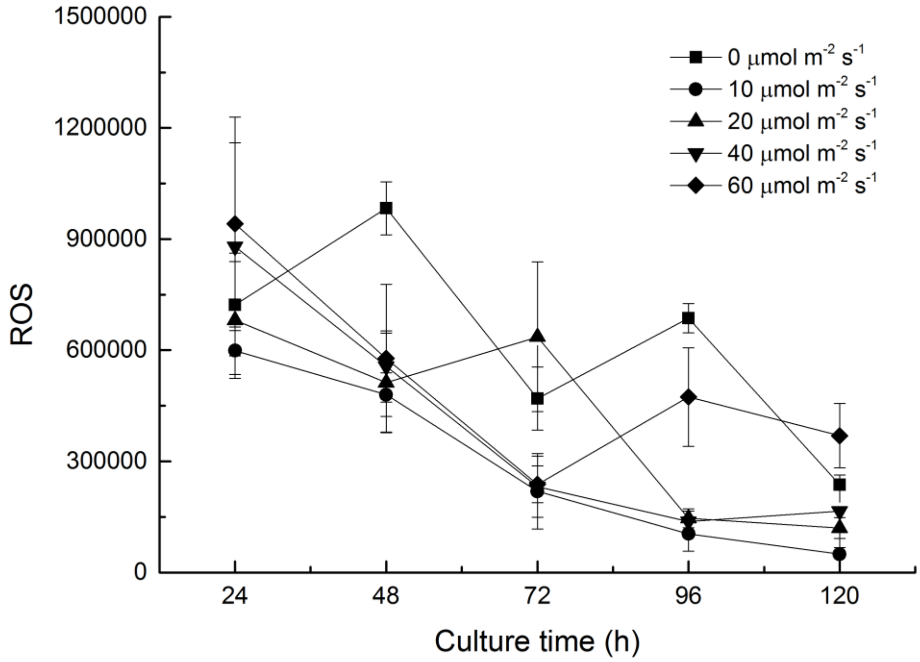

Fig. S1 Effect of light intensity on ROS level of *N. laevis*. Values are mean ± SD of at least three independent experiments.

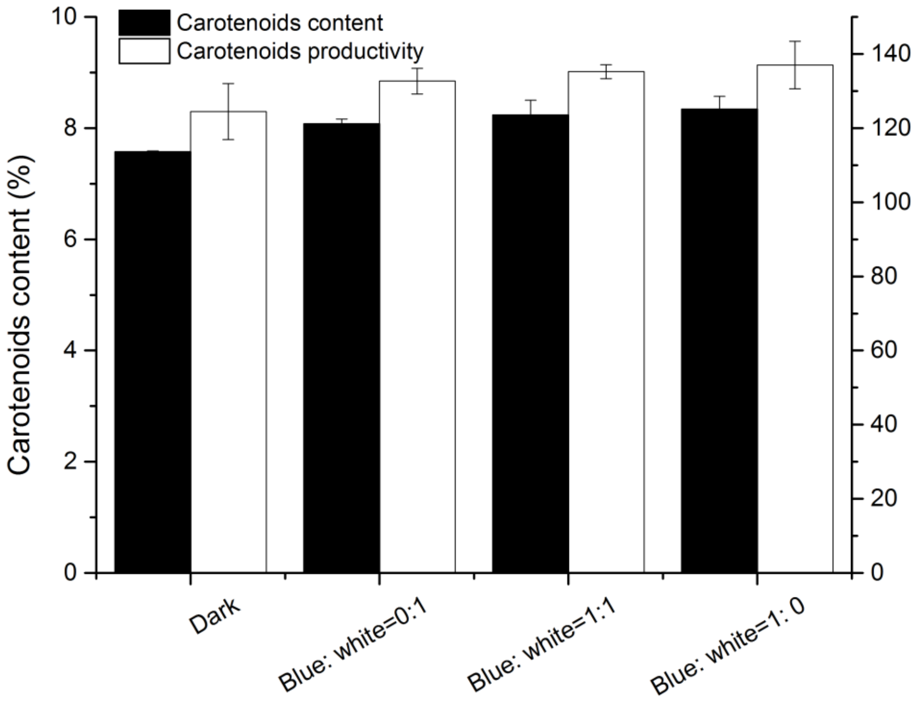

Fig. S2 Effect of light quality on carotenoids content of *N. laevis*. Values are mean ± SD of at least three independent experiments.
